# Food Frequency Questionnaires Validated in Brazil: A Scoping Review

**DOI:** 10.1111/jhn.70190

**Published:** 2026-01-05

**Authors:** Acsa Nara A. Brito Barros, Maria Luisa N. Felipe, Maria Fernanda S. Bezerra, Lucia Leite‐Lais, Lucia Fátima Campos Pedrosa

**Affiliations:** ^1^ Graduate Program in Health Sciences Federal University of Rio Grande do Norte Natal Brazil; ^2^ Graduate Program in Nutrition Federal University of Rio Grande do Norte Natal Brazil; ^3^ Nutrition Undergraduate Course Federal University of Rio Grande do Norte Natal Brazil; ^4^ Department of Nutrition Federal University of Rio Grande do Norte Natal Brazil

## Abstract

**Objective:**

To map food frequency questionnaires (FFQs) validated for the Brazilian population.

**Methods:**

This review was conducted according to the Preferred Reporting Items for Systematic Reviews and Meta‐Analyses for Scoping (PRISMA‐ScR) guidelines and the Joanna Briggs Institute (JBI). Studies that validated FFQs specifically for the Brazilian population were included. These instruments assessed the intake of energy, nutrients, foods and food groups. Review articles and studies that did not focus on FFQ validation for Brazil or its populations were excluded. A search of articles published up to June 2025 was conducted in the PubMed/MEDLINE, LILACS, Embase, Web of Science (ISI), Scopus and Google Scholar databases.

**Results:**

A total of 69 articles were identified, dating back to the late 1990s: Southeast (*n* = 35), South (*n* = 12), Northeast (*n* = 10), Central‐West (*n* = 4), North (*n* = 1) and studies covering two or more regions or populations (*n* = 7). The FFQs were validated in populations of children and adolescents (*n* = 18), adults and older adults (*n* = 36) and women (*n* = 15). These studies differed in terms of food list size, portion size and the number of frequency categories.

**Conclusion:**

Compiling and disseminating FFQs validated in Brazil will improve their use in clinical practice and provide a valuable resource for designing future research on food consumption.

## Summary

- Validated FFQs are available for a wide range of age groups.
- Research on food consumption is unevenly distributed across Brazil.
- There is a need for FFQs that reflect the unique eating habits in certain regions.

## Graphical abstract

Food frequency questionnaires (FFQs) validated for the Brazilian population, according to regions, dietary intake and target populations.

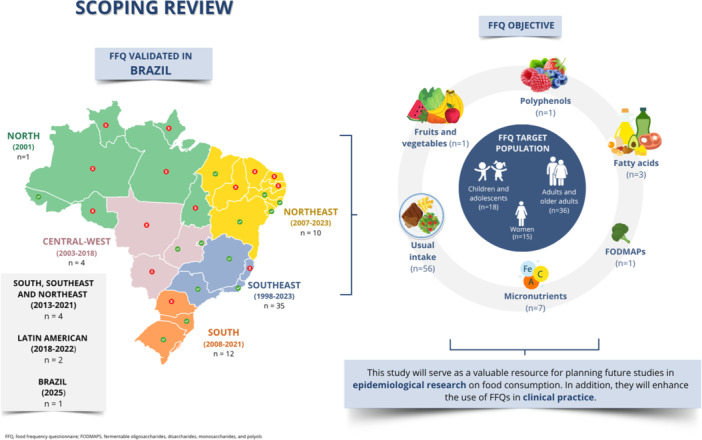

## 1 Introduction

Understanding the relationship between diet and health outcomes is essential in epidemiological studies, making food frequency questionnaires (FFQ) a primary method for estimating food consumption worldwide [1].

An FFQ consists of a list of foods that guides the respondents in recording their frequency of consumption of each food over a specified period [2]. It can be qualitative (excluding data on portion size), semi‐quantitative (including a reference portion size) or quantitative (with open or closed questions about the amount and variable portion sizes consumed) [2]. The FFQ is an objective, low‐cost and easy‐to‐administer instrument. However, its fixed list of foods and reliance on the respondent's memory may lead to inaccuracies or omissions [3].

An FFQ must be carefully developed, considering the study's objectives and specific aspects of the instrument, such as the target population's characteristics, nutrients of interest, list size and type of FFQ (quantitative, qualitative or semi‐quantitative). Furthermore, information about sex, age and cultural and regional factors is essential, as they can influence eating habits [4]. Following development, a validation study is required to compare the FFQ with a reference method [5]. Therefore, developing a new FFQ can be costly and unnecessary when existing questionnaires can be adapted, as long as the instrument adequately matches the characteristics of the study sample [2].

Numerous FFQs have been developed for diverse populations [6, 7, 8, 9, 10, 11, 12, 13, 14, 15]. A narrative review compiled studies that developed and validated FFQs in Brazil until 2013 [9]. Since then, many FFQs have been developed and validated for target populations across different age groups [16, 17, 18] and health conditions [19, 20] in various regions of Brazil. FFQs are crucial tools for assessing food consumption in epidemiological studies. In a country as vast as Brazil (8,516,000 km²), which is divided into five geographic regions (North, Northeast, Central‐West, Southeast and South), dietary habits vary significantly. Therefore, mapping available validated FFQs will assist public health professionals and researchers in selecting appropriate instruments for planning food consumption studies. This review may also encourage the development of new FFQs or the adaptation of existing ones, avoiding unnecessary duplication of effort. To address this, a scoping review was conducted to systematically map the FFQs validated for the Brazilian population and to identify gaps in this knowledge area.

## 2 Methods

This review was conducted according to the Preferred Reporting Items for Systematic Reviews and Meta‐Analyses for Scoping (PRISMA‐ScR) guidelines [19, 20] and the Joanna Briggs Institute (JBI) [21, 22]. The review protocol was registered in the Open Science Framework.

### 2.1 Research Question

This scoping review was designed to answer the question ‘Which FFQs have been validated for the Brazilian population?’ The Population, Concept and Context (PCC) framework was used to construct research questions. The *Population* was defined as nonspecific (open) because we did not restrict the target population of the FFQs to factors such as health status, clinical condition, age, sex or other specific characteristics. The *Concept* was defined as ‘Food Frequency Questionnaires’ that assessed the intake of energy, nutrients (either specific or general), individual foods or food groups. The *Context* was defined as ‘Brazil’, encompassing all regions of the country.

### 2.2 Search Strategy

A literature search was conducted in PubMed/MEDLINE, Embase, LILACS, Web of Science, Scopus and Google Scholar to identify articles published up to June 2025. Keywords in the titles and abstracts of relevant studies, along with the indexed terms of the articles, were used to develop a comprehensive search strategy. The primary keywords used in the search queries were ‘food frequency questionnaire’, ‘validity’ and ‘Brazil’. Additional synonyms, MeSH terms and Emtree terms were incorporated into each query formulation, based on the database being searched. Reference lists of the included studies were reviewed to identify additional relevant studies (Supporting Information: S1).

### 2.3 Eligibility Criteria

Studies were included if they validated FFQs for the Brazilian population and assessed the intake of energy, nutrients, foods and food groups, regardless of publication language or year. Studies involving specific cohorts (e.g., pregnant women, patients with diseases) and regional subsamples were included to reflect the dietary diversity and complexity of food consumption across the country. In multicentre or multinational studies, only those that reported validation results specifically and independently for the Brazilian subsample were considered eligible. Review articles and studies that did not specifically focus on FFQ validation for Brazil or its populations were excluded.

### 2.4 Study Selection

All studies identified through the search were compiled and entered into the Rayyan online application (Qatar, 2022). Duplicates were removed. The titles and abstracts were screened by two independent reviewers according to the inclusion criteria. Full texts of potentially eligible articles were then retrieved and assessed by the same reviewers. Discrepancies between the reviewers were resolved with the input of an additional reviewer.

### 2.5 Data Extraction and Analysis

Data were extracted by two reviewers using a form developed by the authors. The extracted data included the following: (a) study characteristics: author, year, reference, language, region of Brazil where it was conducted, title and study objective; (b) characteristics of the target population, target population, sex, age range and health condition; and (c) characteristics of the FFQ: type (quantitative, semi‐quantitative or qualitative), FFQ objective, number of items, assessment period, portion size availability, number of frequency intake categories, number and type of nutrients/foods/food groups assessed, method of list development, presence of visual support, mode of administration, validation method and authors' conclusions (Supporting Information: S2).

The extracted results were described according to the region of the country where the FFQ was validated, characteristics of the target population and age range of the target population. Validation studies referring to the same FFQ were counted separately, as they were validated for populations with different characteristics or with the aim of assessing different nutrients or food groups.

## 3 Results

**Figure 1:**
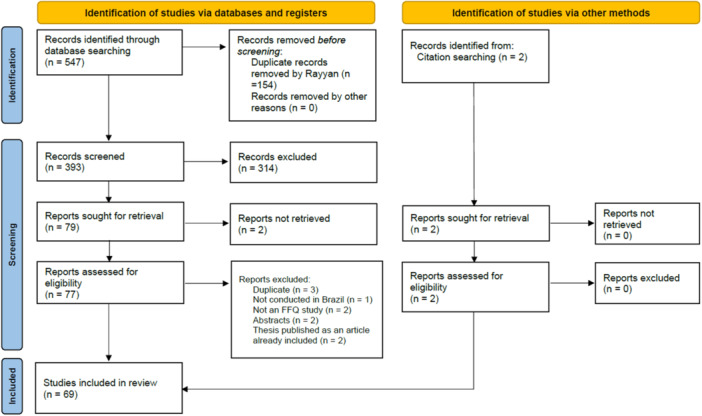
PRISMA‐ScR flow diagram of the study selection process. *Source:* Page MJ et al. BMJ 2021;372:n71. doi:10.1136/bmj.n71.

The database search identified 547 records, with 393 remaining after duplicate removal. After screening titles and abstracts, 77 articles were assessed for eligibility. Ten articles were excluded for not meeting the inclusion criteria, while two additional studies were included from the reference list searches, resulting in 69 eligible articles (Figure 1).

### 3.1 FFQs Validated by Region in Brazil

**Figure 2:**
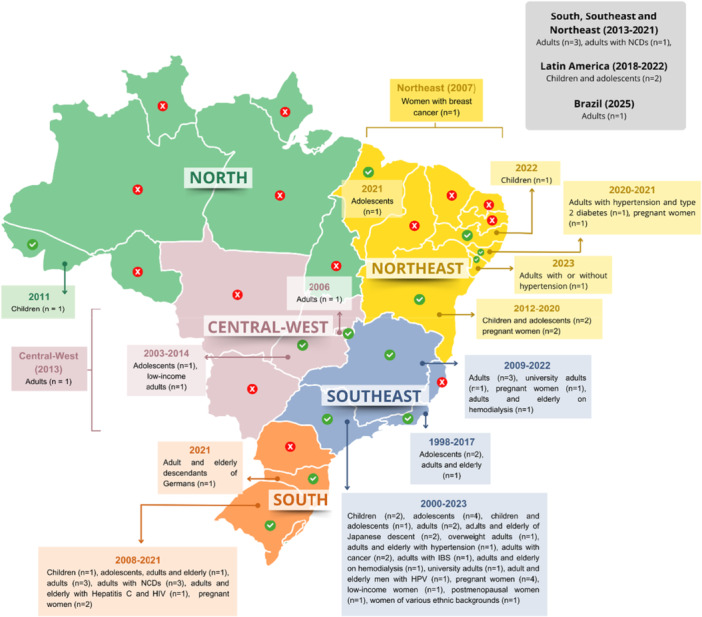
Distribution of food frequency questionnaires (FFQs) validated for the Brazilian population, according to region, number of instruments and target population. The grey text box highlights FFQs validated for multiple regions. The red icons on the states indicate regions without FFQs. HIV, human immunodeficiency virus; HPV, human papillomavirus; IBS, irritable bowel syndrome; n, number of FFQs; NCD, noncommunicable chronic diseases.

Validated FFQs were identified from all Brazilian regions. The Southeast region produced the highest number of studies (*n* = 35), followed in descending order by the South (*n* = 12), Northeast (*n* = 10), Central‐West (*n* = 4) and North (*n* = 1) regions. Seven additional studies covered multiple regions or the entire Brazilian population (Figure 2).

Certain states lacked region‐specific FFQs. In the Southeast, South and Northeast regions, studies targeted populations across various age groups, including children, adolescents, adults and older adults, as well as individuals with distinct health conditions. Fewer studies were conducted in the Central‐West and North regions (Figure 2).

In addition to FFQs developed for different age groups, FFQs targeting Brazilians of various ethnicities and individuals with diverse clinical conditions were identified. These included individuals with hepatitis C and human immunodeficiency virus, noncommunicable chronic diseases, pregnancy, cancer, haemodialyses, irritable bowel syndrome, human papillomavirus and osteoporosis (Figure 2).

### 3.2 Characteristics of Validated FFQs

Among the 69 validated FFQs, 18 FFQs were validated for children and adolescents, 36 for adults and older adults and 15 for women. Regarding the FFQ type, 33 were semi‐quantitative, 32 were quantitative and four were qualitative. The objective was to assess the usual dietary intake (*n* = 56) and specific nutrients, bioactive compounds or food groups, namely, fatty acids (*n* = 3), micronutrients (*n* = 7), polyphenols (*n* = 1), fruits and vegetables (*n* = 1) and fermentable oligosaccharides, disaccharides, monosaccharides and polyols (*n* = 1). The list sizes varied from 7 to 161 items. The assessment period for the FFQs ranged from 1 to 12 months (Table 1).

**Table 1:** Food frequency questionnaires validated in Brazil.

Author	Author of original FFQ	Region	Target population of the validation study	Type	FFQ evaluation target	Number of food items on the list	Number of frequency categories for food consumption	Portion size	Assessment period
Children and adolescents									
Slater et al. (2003) [23]	Same authors	Southeast	Individuals aged 14 to 18 years	Semi‐quantitative	Usual intake	76	Seven	Medium	6 months
Voci, Enes, and Slater (2008) [24]	Slater et al. (2003) [23]	Southeast	Individuals aged 11 to 15 years	Semi‐quantitative	Usual intake	94	Seven	Medium	6 months
Fumagalli et al. (2008) [25]	Cardoso et al. (2001) [26]	Southeast	Individuals aged 5 to 10 years	Quantitative	Usual intake	75	No information	Small, medium or large	No information
Araujo, Yokoo, Pereira (2010) [27]	Same authors	Southeast	Individuals aged 12 to 19 years	Semi‐quantitative	Usual intake	90	Eight	Medium	6 months
Slater et al. (2010) [28]	Same authors	Southeast	Individuals aged 14 to 18 years	Semi‐quantitative	Usual intake	94	Seven	Medium	6 months
Scagliusi et al. (2011) [29]	Tomita et al. (2009) [30]	North	Individuals aged 6 to 9 years	Semi‐quantitative	Usual intake	50	Eight	Medium	1 month
Pino and Friedman (2011) [31]	Slater et al. (2003) [23]	South	Individuals aged 6 to 10 years	Semi‐quantitative	Usual intake	90	Seven	Small, medium, large or extra‐large	12 months
Matos et al. (2012) [32]	Same authors	Northeast	Individuals aged 4 to 11 years	Qualitative	Usual intake	98	No frequency category is defined	No portion size is defined	No information
Martinez et al. (2013) [33]	Same authors	Southeast	Individuals aged 15 to 18 years	Qualitative	Usual intake	50	Seven	No portion size is defined	12 months
Marques et al. (2014) [34]	Fornés and Stringhini (2005) [35]	Central‐West	Individuals with type 1 diabetes aged 10 to 18 years	Quantitative	Usual intake	106	Nine	Small, medium and large	3 months
Mascarenhas et al. (2016) [36]	Same authors	Northeast	Individuals aged 11 to 17 years	Semi‐quantitative	Usual intake	97	Six	No portion size is defined	6 months
Brito et al. (2017) [37]	Araújo et al. (2008) [38]	Southeast	Individuals aged 11 to 18 years	Semi‐quantitative	Usual intake	93	No information	Medium	3 months
Hinnig et al. (2018) [39]	Hinnig et al. (2014) [40]	Southeast	Individuals aged 7 to 10 years	Quantitative	Usual intake	76	Eight	Small, medium and large	3 months
Rendo‐Urteaga et al. (2020) [41]	Saravia et al. (2018) [42]	Latin American	Individuals aged 3 to 18 years	Semi‐quantitative	Usual intake	68−69	Nine	Medium	3 months
Bogea et al. (2021) [16]	Schneider et al. (2016) [43]	Northeast	Individuals aged 18 to 19 years	Semi‐quantitative	Usual intake	89	Seven	Medium, larger than medium or smaller than medium	No information
Hillesheim et al. (2021) [17]	Ribeiro and Cardoso (2002) [44]	Southeast	Individuals aged 9 to 13 years	Quantitative	Usual intake	71	No information	Small, medium and large	1 month
Jurema‐Santos et al. (2022) [45]	Same authors	Northeast	Individuals aged 7 to 10 years	Quantitative	Usual intake	81	Three	Small, medium, large or extra‐large	12 months
Collese et al. (2022) [46]	Saravia et al. (2018) [42]	Latin American	Individuals aged 3 to 17 years	Semi‐quantitative	Usual intake of iron	67−69	Nine	Medium	3 months
Adults and older adults									
Sichieri and Everhart (1998) [47]	Same authors	Southeast	Individuals	Quantitative	Usual intake	73	Six	No portion size is defined	No information
Salvo and Gimeno (2002) [48]	Same authors	Southeast	Individuals with overweight (BMI > 25 kg/m²) aged 18 to 60 years	Semi‐quantitative	Usual intake	90	Three	Small, medium, large or extra‐large	1 month
Fornes, Stringhini, and Elias (2003) [49]	Same authors	Central‐West	Low‐income individuals aged 18 to 60 years	Semi‐quantitative	Usual intake	127	Nine	No portion size is defined	6 months
Ribeiro et al. (2006) [50]	Sichieri and Everhart (1998) [47]	Central‐West	Individuals aged 35 to 50 years	Semi‐quantitative	Usual intake	52	Eight	No information	No information
Matarazzo et al. (2006) [51]	Boffetta, Brennan, and Herrero (1998) [52]	Southeast	Individuals with head and neck cancer aged 37 to 81 years	Quantitative	Usual intake	26	No information	No information	No information
Zanolla et al. (2009) [53]	Same authors	South	Individuals aged 20 to 69 years	Quantitative	Usual intake	127	No frequency category is defined	No portion size is defined	1 month
Crispim et al. (2009) [54]	Sales et al. (2006) [55]	Southeast	Individuals aged 21 to 59 years	Semi‐quantitative	Usual intake	58	Ten	Five portion sizes (A, B, C, D, E)	4 months
Ferreira‐Sae et al. (2009) [56]	Same authors	Southeast	Individuals with hypertension aged 18 to 85 years	Semi‐quantitative	Usual intake of sodium	44	Seven	Medium	12 months
Henn et al. (2010) [57]	Same authors	South	Individuals aged 12 to 90 years	Quantitative	Usual intake	135	No frequency category is defined	No portion size is defined	12 months
Lameza (2010) [58]	Same authors	Southeast	Individuals with colorectal cancer aged 22 to 91 years	Quantitative	Usual intake	110	Four	Small, medium or large	6 months
Teixeira et al. (2011) [59]	Fisberg et al. (2008) [60]	Southeast	Individuals infected with the HPV virus aged 18 to 70 years	Quantitative	Usual intake	54	Four	Small, medium, large or extra‐large	12 months
Machado et al. (2012) [61]	Zanolla et al. (2009) [53]	South	Individuals aged 20 to 69 years	Quantitative	Usual intake	120	No frequency category is defined	No portion size is defined	4 months
Pakseresht et al. (2012) [62]	Sharma et al. (2009) [63]	Southeast	Descendants of Japanese aged 40 to 75 years	Quantitative	Usual intake	161	Eight	No portion size is defined	No information
Silva et al. (2013) [64]	Ferreira et al. (2010) [65]	Central‐West	Individuals aged 20 to 50 years	Quantitative	Usual intake	81	Eight	Small, medium or large	6 months
Sarmento et al. (2013) [66]	Sarmento et al. (2013) [67]	South	Individuals with type 2 diabetes aged 30 to 80 years	Quantitative	Usual intake	98	Four	Small, medium, large or extra‐large	12 months
Komatsu et al. (2013) [68]	Carvalho et al. (2010) [69]	Southeast	University students	Quantitative	Usual intake	70	Eleven	Small, medium or large	No information
Molina et al. (2013) [70]	Sichieri and Everhart (1998) [47]	South, Southeast and Northeast	Individuals with NCDs aged 35 to 74 years	Semi‐quantitative	Usual intake	114	Eight	Medium	12 months
Bonatto et al. (2014) [71]	Zanolla et al. (2009) [53]	South	Individuals with ≥ BMI 30 kg/m² aged 20 to 69 years	Semi‐quantitative	Usual intake	120	No frequency category is defined	No portion size is defined	1 months
Selem et al. (2014) [72]	Fisberg et al. (2008) [60]	Southeast	Individuals aged 20 years or older	Quantitative	Usual intake	60	Eleven	Small, medium, large and extra‐large	12 months
Mannato et al. (2015) [73]	Sichieri and Everhart (1998) [47]	South, Southeast and Northeast	Individuals aged 35 to 54 years	Semi‐quantitative	Usual intake	76	Eight	Medium	12 months
Pereira et al. (2016) [74]	Molina et al. (2013) [70]	South, Southeast and Northeast	Individuals aged 51 to 85 years	Semi‐quantitative	Usual intake of sodium and potassium	114	Eight	No information	12 months
Zanolla et al. (2017) [75]	Same authors	South	Individuals with coinfection of hepatitis C and HIV aged 18 years or older	Quantitative	Habitual diet	75	Eight	No portion size is defined	1 month
Silva et al. (2017) [76]	Segheto et al. (2015) [77]	Southeast	Individuals aged 20 to 59 years	Quantitative	Usual intake of fatty acids	95	Thirteen	Small, medium, large and extra‐large	100 days
Lopes et al. (2017) [78]	Lopes et al. (2010) [79]	Southeast	Individuals with aged 49 to 65 years	Semi‐quantitative	Usual intake of fruits and vegetables	FFQ: 23 Short FFQ: 7	Nine Seven	Medium	No information
Ataide‐Silva et al. (2020) [80]	Same authors	Northeast	Individuals with hypertension and type 2 diabetes aged 20 to 60 years	Quantitative	Usual intake	126	No information	Small, medium and large	No information
Rossato et al. (2021) [81]	Same authors	South	Individuals with prehypertension or mild hypertension aged 30 to 70 years	Qualitative	Usual intake	40	No frequency category is defined	Medium	1 week and 1 month
Vaz et al. (2021) [82]	Schneider et al. (2016) [43]	South	Individuals with 22 years	Semi‐quantitative	Usual intake	92	Eight	Small, medium and large	No information
Chiarelli et al. (2021) [83]	Molina et al. (2013) [70]	South	Descendants of Germans residing in Brazil with a mean age of 54.1 (15.1) years	Semi‐quantitative	Usual intake	116	Nine	No information	12 months
Azarias et al. (2021) [84]	Same authors	Southeast	University students with a mean age of 34.4 (8.6) years	Quantitative	Usual intake	144	Ten	Small, medium and large	1 year
Yamashita et al. (2021) [85]	Same authors	Southeast	Individuals with irritable bowel syndrome aged 20 to 59 years	Semi‐quantitative	Usual intake of FODMAPs	54	Eleven	Medium	3 months
Martinez et al. (2021) [86]	Molina et al. (2013) [70]	South, Southeast and Northeast	Individuals aged 35 to 74 years	Semi‐quantitative	Usual intake	114	Eight	Medium	12 months
Wendling et al. (2022) [87]	Same authors	Southeast	Individuals in haemodialyses treatment over 18 years old	Qualitative	Usual intake	135	No information	No portion size defined	12 months
Souza et al. (2023) [88]	Santo et al. (2021) [89]	Northeast	Individuals with aged 18 to 60 years	Semi‐quantitative	Usual intake of sodium	55	Eight	1/2 portion, 1 portion, 2 portions and more portions	6 months
Muniz et al. (2023) [90]	Same authors	Southeast	Individuals in haemodialyses treatment over 18 years old	Quantitative	Usual intake of potassium	85	No information	Small, medium, large and extra‐large	1 week
Muniz et al. (2023) [91]	Cardoso and Stocco (2000) [92]	Southeast	Individuals aged 20 to 40 years	Semi‐quantitative	Usual intake	97	Seven	Small, medium, large	12 months
Frade et al. (2025) [93]	Same authors	Brazil	Individuals aged 18 years or older	Semi‐quantitative	Usual intake according to the NOVA Classification	99	Ten	Seven portion sizes based on a medium portion	12 months
Women									
Vian et al. (2015) [94]	Same authors	South	Pregnant women with a mean age of 27 (6.7) years	Quantitative	Usual intake of polyphenols	52	Eight	Medium, larger than medium or smaller than medium	15 days
Cardoso et al. (2001) [26]	Cardoso and Stocco (2000) [92]	Southeast	Descendants of Japanese aged 21 to 62 years	Quantitative	Usual intake	31	Four	Small, medium, large and extra‐large	12 months
Lima et al. (2007) [95]	Lima, Fisberg and Slater (2003) [96]	Northeast	Women with breast cancer aged 25 to 80 years	Quantitative	Usual intake	68	Four	Small, medium, large and extra‐large	12 months
Giacomello et al. (2008) [97]	Sichieri and Everhar (1998) [47]	South	Pregnant women aged 15 to 42 years	Quantitative	Usual intake	80	Eight	No portion size is defined	12 months
Pereira et al. (2009) [98]	Same authors	Southeast	Women with osteoporosis aged 51 to 85 years	Quantitative	Usual intake of energy, protein, phosphorus and magnesium	60	Seven	Small, medium and large	1 month and 3 weeks
Ishihara et al. (2009) [99]	Iwasaki et al. (2009) [100]	Southeast	Women of diverse ethnic origins aged 30 to 70 years	Semi‐quantitative	Usual intake	118	Eight	Small, medium and large	12 months
Cardoso, Tomita, and Laguna (2010) [101]	Cardoso et al. (2001) [26]	Southeast	Low‐income women aged 21 to 65 years	Quantitative	Usual intake	76	No information	No information	12 months
Sartorelli et al. (2012) [102]	Oliveira et al. (2010) [103]	Southeast	Pregnant women aged 18 to 35 years	Quantitative	Usual intake of omega‐3, omega‐6 and trans fatty acids.	85	Four	Small, medium, large and extra‐large	Confirmed pregnancy date until the date of administration
Barbieri et a. (2013) [104]	Oliveira et al. (2010) [103]	Southeast	Pregnant women aged 18 to 35 years	Quantitative	Usual intake	85	Four	Small, medium, large or extra‐large	8 months
Barbieri et al. (2015) [105]	Oliveira et al. (2010) [103]	Southeast	Pregnant women aged 18 to 35 years	Quantitative	Usual intake	85	No information	No information	Confirmed pregnancy date until the date of administration
Isobe et al. (2017) [106]	Tuma (2005) [107]	Southeast	Pregnant women aged 19 to 39 years	Semi‐quantitative	Usual intake of vitamin A	32	No information	Small, medium and large	30 days
Carvalho et al. (2020) [108]	Same authors	Northeast	Pregnant women aged 18 years or older	Semi‐quantitative	Usual intake of lipids and fatty acids	FFQ: 87 Short FFQ: 27	Thirteen	Medium	No information
Duarte et al. (2020) [109]	Ribeiro et al. (2006) [50], adapted from Sichieri and Everhart (1998) [47]	Southeast	Pregnant women aged 18 years or older	Semi‐quantitative	Usual intake	52	Seven	Medium	No information
Tenório et al. (2021) [110]	Same authors	Northeast	Pregnant women with a mean age of 24.7 (6.4) years	Quantitative	Usual intake	112	No information	Small, medium and large	30 days
Brito et al. (2024) [111]	Same authors	Northeast	Pregnant women aged 18 years or older	Semi‐quantitative	Usual intake	60	Seven	Medium	30 days

Abbreviations: BMI, body mass index; FODMAP, fermentable oligosaccharides, disaccharides, monosaccharides and polyols; HIV, human immunodeficiency virus; NCDs, noncommunicable diseases.

### 3.3 Characteristics of the FFQ Validation Process

During the validation process, 49 studies used 24‐hour dietary recall (24DR) as a comparison method, nine used food records, two used biomarkers and nine applied more than one method (Table 2). Mean comparisons were analysed using *t*‐tests, Mann−Whitney *U* tests and Wilcoxon tests. Correlations between the dietary intake measures were obtained using Pearson's correlation coefficients, Spearman's correlation coefficients, intraclass correlation coefficients and partial correlation coefficients. The agreement was assessed using kappa statistics and Bland−Altman plots (Table 2).

**Table 2:** Methodological characteristics of food frequency questionnaires validation instruments in Brazil.

Author/year	Diet components assessed	Reference method	Statistical analysis conducted	Correlation coefficients (minimum−maximum values)^c^	Authors' conclusion after validation
Sichieri and Everhart (1998) [49]	Energy, macronutrients, fibre and four micronutrients	4‐day 24DR	Pearson's correlation coefficient	0.1−0.55	Results of the food frequency questionnaire and the 24‐DR were correlated to a similar degree as in studies of other populations. Correlation coefficients for most nutrients were highly significant
Cardoso et al. (2001) [29]^a^	Energy, macronutrients, cholesterol, fibres and 15 micronutrients	3‐day 24DR	Pearson's intra‐class correlation, Pearson's correlation coefficients and comparison of quartile ranking	0.11−0.54	This FFQ could be used to classify individuals of Japanese ancestry living in Sao Paulo according to their nutrient intakes, with a known degree of precision and accuracy
Salvo and Gimeno (2002) [50]^a^	Energy and macronutrients	3‐day 24DR	*T*‐test, intraclass correlation coefficient, weighted kappa and Bland−Altman method	0.01−0.21	The FFQ demonstrated a consumption pattern similar to that observed with the 24DR. Therefore, its use is recommended in overweight populations, as its known limitations can be addressed through calibration studies
Slater et al. (2003) [26]	Energy, macronutrients, cholesterol, fibres and four micronutrients	3‐day 24DR	*T*‐test, Pearson's correlation coefficients and comparison of quartile ranking	Females: 0.10−0.72 Males: 0.16−0.91	The FFQ appears to be a potentially reliable tool for categorizing individuals by their past intake levels of most nutrients, with the exception of retinol and iron
Fornes, Stringhini and Elias (2003) [51]^a^	Energy, macronutrients and four micronutrients	6‐day 24DR	Pearson's correlation coefficient and intraclass correlation coefficient	0.21−0.70	The FFQ demonstrated satisfactory reproducibility and acceptable validity
Ribeiro et al. (2006) [52]^a^	Energy, macronutrients, cholesterol, fibres and five micronutrients	3‐day 24DR	Pearson's correlation coefficient and intraclass correlation coefficient	0.32−0.66	The FFQ constitutes a good research instrument for epidemiological studies in the adult population
Matarazzo et al. (2006) [53]^a^	Seven foods and eight food groups	1‐day 24DR	Wilcoxon test, Spearman's correlation coefficients, intraclass correlation coefficient and comparison of quartile ranking	0.05−0.71	The FFQ used in the Latin American Study demonstrated good reproducibility and acceptable validity for estimating dietary intake
Lima et al. (2007) [95]	Energy, macronutrients and two micronutrients	4‐day 24DR	*T*‐test and Pearson's correlation coefficient	0.36−0.67	The FFQ demonstrated satisfactory validity for use in studies on diet and cancer among women in Paraiba
Voci, Enes, and Slater (2008) [27]	Seventeen food groups	2‐day 24DR	Pearson's and Spearman's correlation coefficients, weighted kappa and Bland−Altman method	−0.26−0.78	The FFQ showed good validity for six food groups
Fumagalli et al. (2008) [28]	Energy, macronutrients, cholesterol, fibres and 11 micronutrients	3‐day food record	*T*‐tests, Pearson's correlation coefficients and weighted kappa	0.02−0.69	The FFQ seems to overestimate energy and nutrient intakes in children aged 5−10 years. Calibration studies are needed to define appropriate portion sizes for this group
Giacomello et al. (2008) [97]	Energy, macronutrients, cholesterol, fibres and 19 micronutrients	4‐day 24DR	Pearson's and Spearman's correlation coefficients, linear regression, weighted kappa and Bland−Altman method	0.01−0.43	The FFQ can be a useful tool to assess the dietary intake of pregnant women in epidemiological studies, provided that efforts are made to improve its performance
Zanolla et al. (2009) [55]^a^	Energy, macronutrients and three micronutrients	3‐day food record	Intraclass correlation coefficient, weighted kappa	0.44−0.83	The FFQ showed good reproducibility and reasonable relative validity
Crispim et al. (2009) [56]	Energy, macronutrients and four micronutrients	4‐day 24DR	*T*‐test, Wilcoxon test and Pearson's correlation coefficient	0.33−0.76	The FFQ showed acceptable performance in assessing the usual dietary intake of most nutrients in the studied population
Ferreira‐Sae et al. (2009) [58]^a^	Sodium	1‐day 24DR, 3‐day food record and biomarker	Mann−Whitney *U* test, Spearman's correlation coefficients and weighted kappa	0.19−0.31	The FFQ demonstrated satisfactory validity and reliability and can serve as an important complementary tool for assessing sodium intake among Brazilian hypertensive individuals
Pereira et al. (2009) [98]	Energy, macronutrients and three micronutrients	3‐day food record	*T*‐test, Pearson's correlation coefficients, comparison of quartile ranking and Bland−Altman method	0.50−0.87	The FFQ for elderly women with osteoporosis is highly acceptable and represents an accurate method for assessing nutrient intakes in this population, making it suitable for use in large‐scale or clinical studies
Ishihara et al. (2009) [99]^a^	Energy, macronutrients, cholesterol, fibres isoflavones, 14 micronutrients and 16 food groups	4‐day food record	Spearman's correlation coefficients	−0.06−0.76	The validity of the FFQ for estimating the intake of selected nutrients among Brazilian women of diverse ethnic backgrounds was moderately high
Araujo, Yokoo, and Pereira (2010) [30]	Energy, macronutrients, fibres and three micronutrients	3‐day food record	Pearson's correlation coefficients, comparison of quartile ranking, weighted kappa and Bland−Altman method	0.33−0.46	The FFQ is an appropriate tool for ranking adolescents according to their energy and nutrient intake
Slater et al. (2010) [31]	Five carotenoids, fruits and vegetables	2‐day 24DR and biomarker	*T*‐test, Pearson's correlation coefficient, partial correlation coefficient	0.21−0.37	The FFQ is an accurate tool for estimating carotenoid, fruit and vegetable intake among adolescents
Henn et al. (2010) [59]	Energy, macronutrients, cholesterol, fibres and seven micronutrients	2‐day 24DR	Pearson's correlation coefficients and comparison of quartile ranking	0.16−0.84	The FFQ showed fair relative validity for adolescents and adults and may be used to study the dietary determinants of obesity and non‐transmissible diseases in epidemiological surveys
Lameza (2010) [60]^a^	Energy, protein, fatty acids, fibres and six micronutrients	3‐day 24DR	*T*‐test, Mann−Whitney *U* test, Pearson's and Spearman's correlation coefficients, comparison of tercile ranking and weighted kappa	0.31−0.62	The FFQ showed adequate performance in classifying individuals according to their habitual intake
Cardoso, Tomita, and Laguna (2010) [101]^a^	Energy, macronutrients, cholesterol, fibres and 13 micronutrients	3‐day 24DR	Pearson's correlation coefficients, intraclass correlation coefficient, Bland−Altman, joint classification analysis and weighted kappa	−0.003−0.75	The FFQ is accurate in assessing nutrient intake at a group level
Scagliusi et al. (2011) [32]	Energy, macronutrients, cholesterol, fibres and 13 micronutrients	2‐day 24DR	Pearson's correlation coefficients, comparison of quintile ranking and Bland−Altman method	−0.03−0.93	The FFQ developed to evaluate the diet of schoolchildren living in the Brazilian Western Amazon proved effective in adequately ranking most nutrient intakes
Pino and Friedman (2011) [34]	Energy, macronutrients, sucrose, cholesterol, fibres and eight micronutrients	3‐day 24DR	Pearson's and Spearman's correlation coefficients, weighted kappa and Bland−Altman method	0.26−0.81	The FFQ was found to underestimate some parameters and overestimate others. It does not demonstrate the necessary relative validity to accurately classify the intake levels of schoolchildren
Teixeira et al. (2011) [61]^a^	Energy, macronutrients, cholesterol, fibres and 10 micronutrients	3‐day 24DR	Pearson's and Spearman's correlation coefficients, intraclass correlation coefficients, weighted kappa and Bland−Altman method	0.05−0.57	The FFQ demonstrated good reproducibility and acceptable validity
Matos et al. (2012) [35]	Energy, macronutrients, cholesterol, fibres and three micronutrients	1‐day 24DR	Pearson's correlation coefficient, weighted kappa and Bland−Altman method	0.14−0.29	The FFQ showed satisfactory validity for use in studies involving children and adolescents
Machado et al. (2012) [63]^a^	Nineteen food groups	3‐day 24DR	Spearman's correlation coefficient, comparison of tertile ranking and weighted kappa	0.53−0.85	The food group‐based FFQ is a suitable alternative for assessing dietary habits, offering the advantage of a shorter food item list
Pakseresht et al. (2012) [64]	Energy, macronutrients, fibres, ethanol, methionine, three carotenoids and nine micronutrients	4‐day food record	Pearson's correlation coefficient, comparison of quartile ranking, weighted kappa and Bland−Altman method	0.02−0.49	The FFQ demonstrated reasonable validity for assessing the daily intake of several dietary nutrients
Sartorelli et al. (2012) [102]	Omega 3, omega 6 and trans fatty acids	3‐day 24DR and biomarker	Pearson's correlation coefficient and comparison of quartile ranking	−0.001−0.57	The tested FFQ was found to be an accurate method for estimating the percentage of fat derived from linolenic acid, docosahexaenoic acid, total omega‐3, arachidonic acid and trans fatty acids
Martinez et al. (2013) [36]^a^	Energy, macronutrients, cholesterol, fibres, 11 micronutrients and eight food groups	4‐day 24DR	*T*‐test, Pearson's correlation coefficient, intraclass correlation coefficient, comparison of tertile ranking and weighted kappa	0.13−0.88	The validity and reproducibility of the FFQ were satisfactory for most variables
Silva et al. (2013) [66]^a^	Energy, macronutrients, cholesterol, fibres and five micronutrients	2‐day 24DR	*T*‐test, Pearson's correlation coefficient, intraclass correlation coefficient, weighted kappa	0.32−0.51	The FFQ showed good reliability and moderate validity for most nutrients based on classification into quartiles of energy and nutrient intake
Sarmento et al. (2013) [68]^a^	Energy, macronutrients, fibres, glycemic index and glycemic load	3‐day weighed food record and biomarker	*T*‐test, Wilcoxon test, Pearson's correlation coefficient and Bland−Altman method	0.19−0.68	The FFQ was valid and precise to assess the usual diet of patients with type 2 diabetes mellitus, according to its validity and reproducibility
Komatsu et al. (2013) [70]	Energy, macronutrients and fibres	3‐day food record	*T*‐test, intraclass correlation coefficient, weighted kappa and Bland−Altman method	0.22−0.43	The FFQ is recommended for assessing the food intake of university students in studies that focus on calorie estimation and aim to classify individuals into intake categories
Molina et al. (2013) [72]^a^	Energy, macronutrients, fibres and eight micronutrients	3‐day 24DR	Intraclass correlation coefficient, comparison of tertile ranking and Bland−Altman method	0.20−0.72	The FFQ demonstrated satisfactory reliability for all nutrients and reasonable validity, particularly for energy, macronutrients, calcium, potassium and vitamins E and C
Barbieri et al. (2013) [104]	Energy, macronutrients, cholesterol, fibres and 15 micronutrients	3‐day 24DR	Pearson's correlation coefficient, comparison of quartile ranking and weighted kappa	−0.10−0.58	The FFQ is a valuable tool for evaluating nutrient intake patterns during pregnancy
Marques et al. (2014) [37]^a^	Energy, macronutrients, cholesterol, fibre and four micronutrients	4‐day 24DR	*T*‐test, Pearson's correlation coefficient, intraclass correlation coefficient, weighted kappa and Bland−Altman method	0.32−0.75	The FFQ showed an acceptable capacity to correctly classify adolescents with type 1 diabetes according to dietary consumption levels
Bonatto et al. (2014) [73]	Energy, macronutrients, cholesterol, fibre and eight micronutrients	3‐day 24DR and biomarker	Intraclass correlation coefficient, weighted kappa and Bland−Altman method	0.14−0.71	The FFQ demonstrated satisfactory relative validity
Selem et al. (2014) [74]^a^	Energy, macronutrients, fibre, cholesterol and 10 micronutrients	3‐day 24DR	Spearman's correlation coefficient, intraclass correlation coefficient and weighted kappa	0.21−0.74	The FFQ showed good validity for estimating the usual food consumption of adults
Vian et al. (2015) [20]	Polyphenols	2‐day 24DR, 1‐day food record and biomarker	*T*‐test, Pearson's correlation coefficient, intraclass correlation coefficient, weighted kappa and Bland−Altman method	0.36−0.72	The FFQ showed validity for quantifying the consumption of total polyphenols in pregnant women
Mannato et al. (2015) [75]	Energy, macronutrients, fibres and nine micronutrients	3‐day 24DR	Pearson's correlation coefficient, intraclass correlation coefficient and Bland−Altman method	0.17−0.66	The FFQ enables comparisons of nutrient intake and makes it possible to identify relationships between diet, cardiovascular diseases and diabetes
Barbieri et al. (2015) [105]	Twenty food groups	3‐day 24DR	Pearson's correlation coefficient and weighted kappa	0.005‐0.54	The FFQ may not be an adequate dietary method to assess the intake of food groups during pregnancy
Mascarenhas et al. (2016) [39]	Energy, macronutrients, fibre and four micronutrients	3‐day food record	*T*‐test, Pearson's correlation coefficient and weighted kappa	0.27−0.99	The FFQ was considered suitable for collecting information on the dietary intake of adolescents in regions with similar diets
Pereira et al. (2016) [76]	Sodium and potassium	3‐day 24DR and biomarker	Partial correlation coefficient and comparison of quintile ranking	Sodium: 0.37 Potassium: 0.60	The FFQ showed good validity in estimating potassium intake in epidemiological studies
Brito et al. (2017) [40]	Energy, macronutrients, cholesterol and five micronutrients	3‐day 24DR	Spearman's correlation coefficient, weighted kappa and Bland−Altman method	0.27−0.49	The FFQ showed acceptable relative validity for categorizing adolescents according to gradients of food consumption
Zanolla et al. (2017) [77]	Energy, macronutrients, fibre and 15 micronutrients	3‐day 24DR	Pearson's correlation coefficient and weighted kappa	0.35−0.81	The FFQ showed satisfactory relative validity for most nutrients
Isobe et al. (2017) [106]	Vitamin A	Biomarker	Pearson's correlation coefficient and weighted kappa	0.48	The accuracy of the FFQ for estimating vitamin A intake was considered moderate, making it suitable for categorizing pregnant women into consumption categories
Hinnig et al. (2018) [42]^a^	Energy, macronutrients, cholesterol and 13 micronutrients	3‐day 24DR	*T*‐test and Wilcoxon test, intraclass correlation coefficients, weighted kappa and Bland−Altman method	0−0.37	The FFQ was not found to be valid for assessing the usual diet of children aged 7−10 years in São Paulo (Southeast region) over a month
Silva et al. (2018) [78]	Energy, total lipids and seven fatty acids	2‐day 24DR and biomarker	Paired *t*‐test, Pearson's correlation coefficient and weighted kappa	0.06−0.90	The FFQ was considered an adequate method for estimating habitual intake of total fat, linoleic acid and linolenic acid
Lopes et al. (2017) [80]	Fruits and vegetables	2‐day 24DR	Wilcoxon test, Spearman's correlation coefficient and weighted kappa	FFQ: 0.24−0.34 Short FFQ: 0.28−0.43	None of the tested methods accurately assessed fruit and vegetable consumption. However, the brief FFQ was considered a suitable tool for investigating fruit consumption
Rendo‐Urteaga et al. (2020) [44]^a^, ^b^	Twelve food groups	3‐day 24DR	Wilcoxon test, Pearson's and Spearman's correlation coefficients, intraclass correlation coefficient and weighted kappa	0.17−0.61	The FFQ showed slight to moderate validity for almost all food groups and can be used to classify the intake of individuals within a population
Ataide‐Silva et al. (2020) [82]^a^	Energy, macronutrients, fibres and eight micronutrients	3‐day 24DR	Paired *t*‐test, Pearson's and Spearman's correlation coefficients and intraclass correlation coefficient	0.06−0.55	The FFQ was considered a good tool for assessing dietary intake, particularly for evaluating energy, macronutrients, calcium and sodium in hypertensive and/or diabetic individuals
Carvalho et al. (2020) [108]	Energy, total lipids and nine types of fatty acids	3‐day 24DR	Pearson's correlation coefficient and Bland−Altman method	FFQ: 0.33−0.62 Short FFQ: 0.09−0.40	The specific FFQ was considered a valid instrument for estimating fatty acid intake among pregnant women
Duarte et al. (2020) [109]	Energy, macronutrients, cholesterol and 13 nutrients	2‐day 24DR	Pearson's correlation coefficient, intraclass correlation coefficient and Bland−Altman method	−0.14−0.34	The FFQ was suggested as a potential tool for assessing dietary intake in pregnant women
Tenório et al. (2021) [110]	Energy, macronutrients, fibre, cholesterol and 16 micronutrients	3‐day 24DR	Pearson's and Spearman's correlation coefficients and the intraclass correlation coefficient	0.02−0.43	The authors concluded that the developed FFQ is a valuable tool for assessing pregnant women's usual food consumption
Bogea et al. (2021) [16]	Macronutrients, cholesterol, C and seven micronutrients	3‐day 24DR	Pearson's correlation coefficient, intraclass correlation coefficient, weighted Kappa and Bland−Altman method	0.06−0.43	The FFQ overestimated the intake of most nutrients, showing acceptable relative validity for some nutrients. It is considered helpful for studies on dietary intake among adolescents
Hillesheim et al. (2021) [17]	Protein, fatty acids, 11 micronutrients and 10 food groups	Biomarker	Mann−Whitney *U* test and Spearman's correlation coefficient	−0.15−0.39	The FFQ was considered valid for classifying Brazilian children and adolescents according to their intake of various nutrients and food groups
Rossato et al. (2021) [19]^a^	Nine foods and 31 food groups	4‐day 24DR	Spearman's correlation coefficient, intraclass correlation coefficient, partial correlation coefficient and Bland−Altman method	1‐month assessment period: 0.11−0.81 1‐week assessment period: 0.10−0.84	Both were considered to have acceptable validity, although the 30‐day FFQ had slightly longer validity
Vaz et al. (2021) [83]	Energy, macronutrients, fibre, cholesterol, 7 micronutrients and 12 food groups	2‐day 24DR	Wilcoxon test, Pearson's correlation coefficient, Lin's concordance correlation coefficient, weighted kappa and Bland−Altman method	0.21−0.66	The FFQ provides a reasonable assessment of usual dietary intake. However, relative validity was weak for specific nutrients and food groups
Chiarelli et al. (2021) [84]^a^	Energy, macronutrients, fibres and 8 micronutrients	3‐day 24DR	Intraclass correlation coefficient, weighted kappa, Bland−Altman method and linear regression	0.00−0.30	The FFQ showed a weak correlation when compared to the reference method
Azarias et al. (2021) [85]^a^	Energy, macronutrients, fibres, 12 micronutrients, added sugars, 8 food groups and 4 food processing categories	4‐day 24DR	Intraclass correlation coefficient and Bland−Altman method	0.25−0.65	The FFQ demonstrated satisfactory validity and can be used for assessing dietary intake
Yamashita et al. (2021) [86]^a^	FODMAPs	3‐day 24DR	Spearman's correlation coefficient, intraclass correlation coefficient, weighted kappa and Bland−Altman method	0.21−0.65	The instrument showed good validity for lactose and limitations for the intake of fructose, polyols and oligosaccharides
Martinez et al. (2021) [87]^a^	Energy, animal protein, plant protein, fructose, five fatty acids and five micronutrients	3‐day 24DR	Intraclass correlation coefficient and comparison of tertile ranking	0.14−0.61	The FFQ showed satisfactory relative validity for six nutrients
Jurema‐Santos et al. (2022) [47]^a^	Energy, macronutrients and four micronutrients	3‐day 24DR	Paired *t*‐test, Spearman's correlation coefficient, intraclass correlation coefficient and Bland−Altman method	0.20−0.75	The FFQ developed was valid and able to assess the local food consumption by children from northeastern Brazil
Collese et al. (2022) [48]^b^	Iron	3‐day 24DR and biomarker	Spearman's correlation coefficient, regression, weighted kappa and Bland−Altman method	Children: 0.85 Adolescents: 0.78	The FFQ demonstrated good validity for classifying children and adolescents according to dietary iron intake
Wendling et al. (2022) [88]^a^	Energy, macronutrients, fibre, cholesterol, 7 micronutrients and 11 food groups	2‐day 24DR	Pearson's correlation coefficient, intraclass correlation coefficient, weighted kappa and Bland−Altman method	0.05−0.51	The FFQ showed moderate validity for calories, nutrients and food groups of clinical interest and may be a valuable tool in epidemiological studies in haemodialyses patients
Souza et al. (2023) [89]^a^	Sodium	3‐day 24DR	Spearman's correlation coefficient, intraclass correlation coefficient, kappa and Bland−Altman method	0.26	The FFQ was not considered valid for assessing sodium intake
Muniz et al. (2023) [91]	Potassium	3‐day food record	Intraclass correlation coefficient and Bland−Altman method	0.66	The FFQ was considered a practical tool in the analysis of potassium intake in haemodialyses patients
Muniz et al. (2023) [92]	Energy, macronutrients, fibre, 13 micronutrients and 21 food groups	2‐day 24DR	Intraclass correlation coefficient and weighted kappa	−0.03−0.62	The authors considered that the food frequency questionnaire has proven useful for estimating the intake of certain nutrients and food groups among the subjects evaluated
Brito et al. (2024) [111]	Energy, macronutrients, fibre and 20 micronutrients	1‐day 24DR	*T*‐test and Pearson's correlation coefficient	−0.15−0.50	The authors concluded that the FFQ was adequately accurate for assessing the food consumption of pregnant women
Frade et al. (2025) [94]^a^	Twelve food groups	2‐day 24DR	Intraclass correlation coefficient, comparison of quintile ranking, weighted kappa and Bland−Altman method	0.61−0.65	The NovaFFQ is a validated instrument for evaluating food intake according to processing level, and is particularly useful for discriminating individuals based on their consumption levels across all NOVA categories

Abbreviations: 24DR, 24‐hour dietary recall; FFQ, food frequency questionnaire.

^a^ Studies with reproducibility analysis.

^b^ Multinational validation studies, where Brazilian data were reported as a subsample.

c The reported correlation coefficient values represent the minimum and maximum values observed in each study.

## 4 Discussion

This review identified FFQs validated across all Brazilian regions since the late 1990s, targeting diverse populations—including children, adolescents, women, adults and older adults—in various clinical settings. The FFQs were semi‐quantitative, quantitative and qualitative, featuring diverse food list sizes, portion sizes and consumption frequency categories.

Most studies were conducted in the Southeast, South and Northeast regions, reflecting the concentration of nutritional epidemiology research groups in these areas, particularly in the Southeast, a leading hub of research and scientific output in Brazil. Conversely, the Northern region, despite its rich cultural diversity, had only one study of FFQs. This highlights the need for increased investment and support for research and training in food and nutrition within the region.

FFQs are used to test epidemiological hypotheses in population studies and investigate the relationship between dietary factors and health outcomes [2]. However, despite their utility, significant regional disparities exist in the distribution of the studies, and many focus on a limited range of health conditions. For example, no FFQs were identified for neurological, liver, gastrointestinal, allergic or food‐sensitivity conditions. Consequently, Brazilian researchers or clinicians interested in assessing habitual dietary intake related to these or other health conditions—whether for the overall diet or a specific nutrient—will need to develop, adapt and validate an FFQ based on an existing one [112, 113, 114, 115, 116, 117, 118]. If the goal is to assess the usual dietary intake, an FFQ validated for sex, age range and geographic location can be used. However, it is essential to consider that the list of foods included in the FFQ should reflect the eating habits of the population studied [2], which may vary depending on health conditions.

The age of the study population also influenced eating habits. Pedraza and Menezes [9] found that, up to 2013, no FFQs were available for children younger than 5 years in Brazil. This review identified two studies for children aged 3 years and older. Given the importance of assessing children's diets for their growth and development [119], there is a notable gap in research on children younger than 3 years. In a review of the quality of FFQ validation studies developed for children aged 12–36 months, Lovell et al. [119] concluded that the instruments analysed were acceptable for estimating calcium, vitamin C and iron intake.

In studies involving women, the majority of FFQs have been validated specifically for use during pregnancy. Bezerra et al. [120] systematically reviewed FFQs validated for pregnant women and found moderate to high validation coefficients (≥ 0.40), indicating that these instruments are reliable for evaluating food intake in this group. For Brazilian researchers who intend to estimate the usual food consumption of women at other stages of life, using FFQs validated for adults is an option.

The number of items on an FFQ affects interview duration, respondent fatigue and accuracy of food intake assessment. In this review, the list of foods in the FFQs validated over the last 10 years ranged from 7 to 161 items. Cade et al. [7] found that FFQs typically range from 5 to 350 items, with an average of 79 items. It is essential to highlight that lists with more than 130 items can cause fatigue, reduce concentration and compromise accuracy without offering greater validity than shorter lists [2]. Depending on the study goals, a smaller list often adequately captures most of the intake of a particular food component [5]. We observed that FFQs with fewer items were successfully used to assess vitamin A [106], fruits and vegetables [78] and fatty acids [108], proving effective for their respective objectives.

Although FFQs are not the most precise method for measuring habitual dietary intake, they are commonly used in prospective studies because of their low‐cost and ease of application [5]. In Brazil, several FFQs have been validated in population‐based studies, such as the FFQ used in the Longitudinal Study of Adult Health (ELSA Brazil), a multicentre cohort investigation involving the country's Northeast, South and Southeast regions [121]. This FFQ, initially developed and validated in 2013 [70, 122], has since been adapted into a shorter version [73] and has undergone further validation for various purposes [74, 86]. Recently, additional FFQs were validated in prospective studies in Brazil [46, 83, 84, 87] and Latin America [41].

Validation of an FFQ is crucial to ensure its accuracy in estimating nutrient and food intake. This process helps prevent erroneous associations between dietary factors and diseases or disease‐related markers [8]. Although biomarkers are the most recommended standards for comparison, owing to their ability to minimize common biases in food consumption assessments, they are not always available. Depending on the study design and population characteristics, repeat food records may be a good option, followed by 24DR and dietary history [123].

In this review, most studies used dietary intake assessment methods as standards for comparison. Cade et al. [7] reported a similar finding, noting that 75% of reviewed studies used 24DR and food records as part of the validation process. Other reviews on FFQs have also highlighted that dietary methods are predominantly used as a reference standard [6, 9, 10, 11, 12, 13, 119].

Regardless of the reference method, the correlation coefficients generally exhibited a linear relationship with the FFQ. In the present review, the coefficients varied among the studies included. The authors typically considered coefficients of 0.4 or higher valid, as recommended. Coefficients below this threshold can weaken the association between diet and diseases [2].

Before using a valid FFQ, researchers should ensure that the instrument aligns with the research objectives and methodological procedures supporting its validation [5]. Both correlation and regression coefficients are useful for testing validity owing to the associations obtained between the different methods of assessing food consumption. Ideally, these coefficients should be used alongside the Bland−Altman method or kappa statistics to evaluate the agreement between the FFQ and other dietary methods. Utilizing multiple statistical approaches enhances the robustness of the validation process [7, 8], a practice observed in most reviewed studies.

A strength of this review is the identification of the limited availability of FFQs in North Brazil, a region characterized by its rich cultural diversity. This highlights the uneven distribution of research focusing on food consumption across the country, leading to fewer validated FFQs that reflect the unique eating habits in specific regions. In addition, there is a shortage of FFQs tailored to specific health conditions, indicating the need for further investigation. Compiling and disseminating validated FFQs across different populations can help prevent redundant developmental efforts and save time and resources. One limitation of this study is the need for further analysis of the quality of the study designs and reference methods used in the validation processes. However, this was not the objective of the review, because the scoping review methodology was inherently comprehensive and exploratory. We also had difficulties with incomplete data on QFA characteristics in these studies.

In conclusion, this review mapped FFQs that have been validated for the Brazilian population. We identified various validated FFQs for children, adolescents, adults, older adults and pregnant women. These FFQs differed in their objectives for assessing habitual diet and specific food components. Since FFQs are essential for epidemiological research on food consumption and require tailored questionnaires for different populations, these results will serve as a valuable resource for planning future studies investigating health−disease relationships and other types of research where habitual dietary intake is a variable of interest.

Additionally, because FFQs provide a quick and practical method for assessing habitual dietary intake in clinical practice, Brazilian health professionals can use these findings to guide the selection of the most appropriate FFQ based on specific interests, considering factors such as sex, age, health conditions and region. In both cases, the absence of FFQs that meet the needs of researchers and clinicians serves as a starting point for the development or adaptation of FFQs to better reflect the reality of each population. Moreover, these results provide an essential informational foundation for future initiatives aimed at creating an online repository of Brazilian FFQs, which could optimize the selection and development of these instruments across different regions of Brazil (Supporting Information: S3).

## Author Contributions

**Acsa Nara A. Brito Barros:** conceptualization, methodology, investigation, writing – original draft. **Maria Luisa N. Felipe:** investigation, writing – original draft. **Maria Fernanda S. Bezerra:** investigation, writing – original draft. **Lucia Leite‐Lais:** conceptualization, methodology, writing – review and editing. **Lucia Fátima Campos Pedrosa:** conceptualization, methodology, writing – review and editing.

## Conflicts of Interest

The authors declare no conflicts of interest.

## Supporting information

S1: Search strategy.

S2: Extraction_form.

S3: Framework.
