# Supplementary material for: Food Frequency Questionnaires Validated in Brazil: A Scoping Review
Source: J Hum Nutr Diet. 2026 Jan 5;39(1):e70190. doi: 10.1111/jhn.70190 (PMC12766558; doi:10.1111/jhn.70190)
Supplement: Supplementary file 1 — S1: Search strategy. [file JHN-39-0-s002.docx]

**Supplementary Material S1**. Search strategy for databases

| **Database** | **Search Terms Combinations with Boolean Operators** |
| --- | --- |
| PubMed | (((("Surveys and Questionnaires"[Mesh] OR (Questionnaires and Surveys) OR (Survey*) OR (Questionnaire Design*)) OR ("Diet Records"[Mesh] OR (Diet* Record))) AND ((FFQ) OR (food frequency questionnaire*))) AND ("Reproducibility of Results"[Mesh] OR (Validity Result*) OR (Validity and Reliability))) AND ("Brazil"[Mesh]) |
| LILACS | ((food frequency questionnaire) OR FFQ) AND (MH:"Reproducibility of Results" OR (Face Validity) OR (Finding Reproducibilit*) OR (Of Result*, Reproducibility) OR (Reliabilities, Test-Retest) OR (Reliability (Epidemiology)) OR (Reliability and Validity) OR (Reliability of Result*) OR (Reliability, Test-Retest) OR (Reproducibility Of Result) OR (Reproducibility of Finding*) OR (Result Reliabilit*) OR (Result Validit*) OR (Result*, Reproducibility Of) OR (Test Retest Reliability) OR (Test-Retest Reliability) OR (Validity (Epidemiology)) OR (Validity and Reliability) OR (Validity of Result*) OR (Validity, Face) OR MH:E05.318.370.725$ OR MH:E05.337.851$ OR MH:N05.715.360.325.685$ OR MH:N06.850.520.445.725$ OR MH:SP5.001.022$) AND (MH:"Brazil" OR MH:Z01.107.757.176$) |
| Embase | #1 'food frequency questionnaire'/exp OR (food frequency questionnaires) OR FFQ  #2 'validation study'/exp OR 'validity'/exp OR (validation studies) OR (validation studies as topic)  #3 'reproducibility'/exp OR (measurement reproducibility) OR (reproducibility of results) OR reproductivity  #4 'Brazil'/exp OR 'Brazilian'/exp OR Brazilians  #1 AND #2 OR #3 AND #4 |
| Google Scholar | #1 food frequency questionnaire OR FFQ  #2 validation OR validity  #3 reproducibility OR reproductivity  #4 'Brazil OR Brazilian OR Brazilians  #1 AND #2 OR #3 AND #4 |
| Scopus | ( ( TITLE-ABS-KEY ( "food frequency questionnaire" ) OR TITLE-ABS-KEY ( ffq ) ) ) AND ( ( TITLE-ABS-KEY ( validation ) OR TITLE-ABS-KEY ( validity ) OR TITLE-ABS-KEY ( reproducibility ) ) ) AND ( ( TITLE-ABS-KEY ( brazil ) OR TITLE-ABS-KEY ( brazilian ) ) ) |
| Web of Science | #1 (ALL=("food frequency questionnaire")) OR ALL=(ffq)  #2 ((ALL=(validation)) OR ALL=(validity)) OR ALL=( reproducibility)  #3(ALL=(brazil)) OR ALL=(brazilian)  #1 AND #2 AND #3 |
