# Supplementary material for: Food Frequency Questionnaires Validated in Brazil: A Scoping Review
Source: J Hum Nutr Diet. 2026 Jan 5;39(1):e70190. doi: 10.1111/jhn.70190 (PMC12766558; doi:10.1111/jhn.70190)
Supplement: Supplementary file 2 — S2: Extraction_form. [file JHN-39-0-s003.docx]

**Supplementary Material S2.** Data extraction form

1. **Data extractor's name**
2. **Extraction date**
3. **Article title**
4. **Author**
5. **Publication year**
6. **DOI/Reference**
7. **Journal**
8. **Brazilian region of study**
9. **Study objective**
10. **Population**
    1. Gender
    2. Age/Age group
    3. Health condition
11. **Characteristics of the FFQ**
    1. Type: quantitative, semiquantitative, or qualitative
    2. FQQ objective
    3. Number of items: number of questions in the instrument
    4. Food portions and frequency: portion sizes consumed and frequency categories used, such as per day, month, or year
    5. Method of administration: self-administered or administered by an interviewer
    6. Validation method: statistical method used for validation
12. **Observations**
