# Supplementary material for: Food Frequency Questionnaires Validated in Brazil: A Scoping Review
Source: J Hum Nutr Diet. 2026 Jan 5;39(1):e70190. doi: 10.1111/jhn.70190 (PMC12766558; doi:10.1111/jhn.70190)
Supplement: Supplementary file 3 — S3: Framework. [file JHN-39-0-s001.docx]

**METADATA FRAMEWORK FOR A BRAZILIAN FFQ REGISTRY**

**Category Metadata Field Description and Purpose**

| **Category** | **Metadata Field** | **Description / Purpose** |
| --- | --- | --- |
| **I. Identification & Scope** | **Instrument Name** | The formal name or acronym of the FFQ. |
|  | **Instrument Purpose** | The primary aim of the instrument (e.g., usual diet, nutrients of interest). |
|  | **Population** | The specific target group for which the FFQ was validated. |
|  | **Region** | The geographical or regional location of the validation study in Brazil. |
|  | **Items** | Total number of food items included in the questionnaire. |
|  | **Frequency Categories** | Number and description of frequency response options. |
|  | **Portion Guidance** | Method used to estimate portion size. |
|  | **Period** | Time frame assessed by the FFQ. |
|  | **Administration Mode** | How the FFQ was administered (e.g., interviewer-administered, self-administered, digital). |
|  | **Visual Aids** | Presence and type of visual aids used (e.g., photographs, models). |
| **II. Instrument Structure** | **Number of Replicates** | Number of repetitions used for the comparator method. |
|  | **Deattenuation** | Whether statistical deattenuation was applied (Yes/No). |
|  | **Energy Adjustment** | Method used to adjust nutrient intake for total energy intake. |
|  | **Primary Statistics** | Main statistical measure used for validation (e.g., correlation coefficients, ICC). |
|  | **Performance Summary** | Brief descriptive summary of the instrument’s validation outcome. |
| **III. Validation Metrics** | **Comparator Method(s)** *(optional, if retained)* | Reference method(s) used for validation (e.g., 24HR, food records, biomarkers). |
|  | **License/Permissions** | Information on copyright or permissions required for use. |
|  | **DOI/Source** | Digital Object Identifier (DOI) or full citation of the original validation paper. |
| **IV. Access & Rights** | **Instrument Availability** | Information on how and where the FFQ can be accessed. |
|  | **Usage Rights** | Terms and conditions for the use of the questionnaire (if applicable). |
